# It is time to target psychological readiness (or lack of readiness) in return to sports after Anterior Cruciate Ligament tears

**DOI:** 10.1186/s40634-023-00657-1

**Published:** 2023-09-20

**Authors:** Anne Gro Heyn Faleide, Eivind Inderhaug

**Affiliations:** 1 Haraldsplass Deaconess Hospital, Ulriksdal 8, 5009 Bergen, Norway; 2 The University of Bergen, Bergen, Norway; 3 Haukeland University Hospital, Bergen, Norway

**Keywords:** Return to sports, Anterior cruciate ligament injury, Psychological readiness, Return-to-sport testing

## Abstract

Functional recovery has for long been the focus of rehabilitation after an Anterior Cruciate Ligament (ACL) injury. It is now increasingly recognized that more attention should be given to patients` mental recovery, their *psychological readiness for returning to sport,* after such an injury. Within this relatively new field of science, clinicians need clarity on when and how psychological factors should be monitored and how inexpedient psychological responses may be addressed during rehabilitation. In this Expert Opinion suggestions on how targeting psychological readiness may improve rehabilitation and return-to-sport evaluations are made based on current evidence—and issues in need of further clarification are addressed.

## Readiness for return to sports after ACL injury

An ACL rupture can be a devastating and potentially career-ending injury affecting young and active people. The overall aim of treatment is restoring knee stability to enable normal daily life functioning and participation at the desired activity levels—without suffering further knee injuries. Depending on the patients target activity level, concomitant injuries, and degree of knee laxity – treatment will often involve surgical reconstruction of the torn ACL. Restoration of neuromuscular function through active rehabilitation can yield acceptable function for some, but increased awareness on the importance of meniscal repair and secondary injury prevention supports a surgical approach in the majority [18, 24, 27, 46, 60].

Even though surgery and rehabilitation seem to result in normal or nearly normal functioning for most patients, many do not regain their previous levels of sports participation. Ardern et al. [6] reported that only 65% of patients returned to pre-injury level of sport after ACLR and no more than 55% were able to return to competitive levels. Such disappointing results are confirmed in later reports [40, 56]. In addition, the threat of subsequent surgery due to meniscal lesions, a graft rupture or a contralateral ACL injury remains significant—further adding to the risk of osteoarthritis development [25, 29, 31, 42, 52]. Therefore, improving the course of treatment and making well-founded return-to-sport decisions are crucial to support patients in reaching their goals of an active lifestyle while safeguarding long-term health and quality of life.

“Functional recovery is the key to success after surgery” has been a mantra among surgeons and therapists aiding the patients’ rehabilitation. A successful recovery has for long been associated with biological healing, restoration of native knee stability and recovery from neuromuscular deficits [12]. Certainly, a lack of functional recovery can leave the patients vulnerable for new injuries, prevent their return to an active lifestyle and cause reduced quality of life [23, 28, 29, 38, 62]. Therefore, a close attention to patients’ progression through their phases of rehabilitation is paramount. Test batteries have been developed to identify and quantify functional deficits allowing a more targeted approach in the latter phase of rehabilitation when patients approach “return to play”. However, several studies have criticized how commonly used test batteries fail to identify patients who will struggle to return safely to sport. It has been hypothesized that some of the explanation can be found in a lack of comprehensiveness in the test batteries—focusing solely on assessment of physical readiness [12, 41, 45, 67].

New evidence emphasize how ACL injuries also have a significant psychological impact and that patients therefore need to achieve a state of “overall readiness” (including mental readiness) for returning safely to sport [8–10, 13, 35, 39, 65]. Yet, implementation of this knowledge to clinical practice is long overdue. The reason for this may be a lack of clarity about how the psychological aspect of readiness should be monitored and addressed in clinical care.

## The impact of psychological readiness

After an ACL injury, patients may experience strong emotions of tension and anxiety, fear of new injuries, reduced confidence, and low motivation [7, 13, 26, 35, 39, 50]. These responses will inevitably affect both rehabilitation and the return-to-sport process [14, 61]. *Psychological readiness* has been found to be an important predictor of patients’ ability to resume sport, in fact a recent study reported it to be *the most important component* of a test battery when it came to predicting a return to pre-injury activity level [8, 10, 17, 22, 23, 39, 48, 67]. Further, there are indications that low psychological readiness might be associated with a higher risk of sustaining a second ACL injury upon returning to sport [44].

An interesting observation is that physical and mental recovery does not always coincide [8, 17, 39, 50]. Patients might have regained sufficient function for sports participation, but still report high levels of fear and low psychological readiness [9]. In such a situation their performance on tests of functional recovery (such as isokinetic strength tests and single-legged hop tests) will have little predictive value in whether they return to sport or not. These patients might end up never attempting a return to play [40, 56]. Conversely, other patients may display no fear and high mental readiness for returning, irrespective of how physically ready they are [13]. These scenarios underline the importance of addressing both physical and psychological recovery—and using a test battery that includes evaluation of a combined physical and psychological readiness is therefore recommended [2, 23, 54].

## Detecting erroneous psychological responses

Several tools for evaluation of psychological responses are available to help clinicians identify patients with mental issues pertaining to their injury and resumption of former athletic activities [20]. The ACL – Return to Sports after Injury (ACL-RSI) scale was developed through literature review and identification of psychological domains hypothesized to be relevant for patients working towards returning to sport after ACLR [65]. Three mental domains (emotions, confidence in performance and risk appraisal) were selected to constitute psychological readiness for resuming sport. The 12-item scale struck a chord with clinicians and researchers, has been translated to and validated in several languages, and is now the dominant measure for psychological readiness in this patient group [1, 11, 21, 30, 33, 36, 55, 57, 58].

There are, however, no evidence-based guidelines to inform clinicians about when psychological readiness should be assessed, how scores should be interpreted or how a *low (or excessively high) readiness* for returning should be addressed in the treatment course [3, 15, 35]. With regard to timing, Ardern et al. [8] found that psychological responses prior to, and 4 months after surgery were associated with patients ability to return to sport one year post-ACLR. Although measuring patients’ preparedness for returning to sport before surgery seems premature, it is suggested that assessment of psychological readiness should start in the relatively early phases of rehabilitation (for example at 4 months) with regular monitoring until the return-to-sport evaluation—often performed at 9 months after surgery [2, 23, 51, 54].

The uncertainty about how ACL-RSI scores should be interpreted during ACL rehabilitation relates to both which values to use for denominating patients who may struggle to return to sport (cut-off values) and to the scale`s responsiveness [51, 59, 64]. Various cut-off values have been reported with a range from 42 to 65 on the 0–100 points scale [8, 23, 48, 54, 63]. At four months, an ACL-RSI score < 56/100 is suggested to differentiate between those who may and those who may not struggle to resume pre-injury level of sports participation one year after surgery [8]. Two studies have reported similar cut-offs at the six months assessment with a score of 60 to 62 points [54, 63]. At nine months after surgery, patients with a score of ≤ 47 may be at risk of not returning to pre-injury sport level two years after ACLR [23]. It might seem counterintuitive that these cut-off values do not gradually increase as rehabilitation and functional performance progresses towards the return-to-sport evaluation. This phenomenon may be due to increased emotional disturbance as patients are approaching a return to sport [47]. Further complicating matters are the indications of problems with responsiveness when the ACL-RSI is used to monitor individuals (as opposed to groups of patients) [59, 64]. At this point, the abovementioned cut-off values should therefore only be used as preliminary indicators when monitoring patients in clinical practice because their predictive value for identifying individuals in need of interventions targeting low mental preparedness needs further investigation.

## Limited knowledge on interventions for low mental readiness

Although we now have the means to detect erroneous psychological responses – there is a lack of evidence on how to target these responses throughout the treatment course [3, 4, 16, 35]. Some recommendations have been made based on available knowledge; *Careful information* about the injury and the content and expected timeframes of rehabilitation is highlighted to reduce fear, increase motivation, and boost confidence [3, 37]. As degree of psychological readiness is associated with functional performance, clinicians may help patients *feel* more ready for challenging their knee by *ensuring adequate physical recovery* [19, 21, 32, 35, 53, 66]. In support of this, patients report that strength training and sport-specific exercises, with a *graded exposure* to challenging tasks, increase confidence that their knee will withstand the pressures and forces that comes with sports [34, 43]. Further, *regular monitoring of functional recovery* throughout rehabilitation is recommended to provide a realistic understanding of how patients are progressing and thereby promote mental recovery [37].

As the association between physical and psychological readiness is only small to moderate [21, 23, 32, 53], there is also a need for developing interventions that more specifically target psychological recovery [4, 16]. Preliminary evidence suggests that visual and guided imagery, goal setting, relaxation techniques, motivational interviewing, cognitive behavioural therapy, and coping modelling may reduce fear [3, 15, 16]. This evidence is, however, still limited, and inconsistent. Acknowledging the need for specific cognitive interventions also underpins how professionals with a background in neuroscience and psychology should be included both in research and treatment of these patients. With a few exceptions, physiotherapists and orthopaedic surgeons have dominated this field, but it now seems timely to expand our understanding by drawing on the experience of other professions.

## Issues in need of clarification

To fully resolve the abovementioned uncertainties, it may be helpful to take a step back before moving forward. A research team including sports psychologists have pointed to ambiguities related to the concept of psychological readiness and therefore proposed the following definition: *“Psychological readiness to RTS [return to sport] after injury reflects an individual’s state of mental preparedness to resume sport-specific activities and likely comprises three dimensions, including cognitive appraisals (confidence, expectations, motivations, risk appraisals, internal or external pressures), affective components (anxiety or fears about re-injury or movement, moods) and behavioural components (approach-avoidance behaviours to demonstrate physical function/neuromuscular control and engage in sport-specific tasks)(*[51]*, p. 7).”*

The definition undoubtedly covers many relevant aspects of feeling mentally prepared for returning to vigorous activity. However, further work is needed to clarify whether certain dimensions are more important than others, whether the psychological responses may take numerous meanings (i.e. confidence in performance and confidence in ability to withstand re-injury) and which elements to include in the construct of psychological readiness [51]. For instance, while motivation and fear of re-injury is included in the proposed definition, others have listed these factors as separate from psychological readiness [5, 51]. A common interdisciplinary understanding of *psychological readiness* would enhance both researchers’ and clinicians’ ability to plan for optimal monitoring of mental recovery and to develop interventions targeting erroneous psychological responses.

Another aspect of readiness that needs to be addressed is whether fear avoidance is a rational and protective mechanism that helps patients avoid further injury when normal knee function has been disrupted. After a traumatic event, and episodes of recurrent instability, patient have painfully experienced how their knee cannot be trusted through strong and fear-provoking stimuli. Indeed, a history of previous injury makes athletes vulnerable to new injuries and being afraid of re-injury is therefore not irrational [37]. For some, the fear and hesitation can lead to positive processes, like reorientation, where they choose to return to a lower level of sport or change to a sport involving less contact or pivoting [37]. Such behaviour will reduce the risk of sustaining further injuries and thereby protect against future osteoarthritis [29, 40]. However, for others, fear can become irrational and thereby have a detrimental effect on the rehabilitation and long-term outcome [37, 51]. These are the individuals in need of targeted psychological interventions. Patients on the other end of the spectrum, displaying high psychological readiness irrespective of muscle strength are probably highly susceptible to new injuries and may need a different treatment approach[49, 68]. Moving forward, exploring such patient profiles would allow a more individualized and targeted rehabilitation.

## Conclusion

Both physical and psychological readiness to return to sport need to be assessed and addressed after an ACL injury. The ACL-RSI is a validated questionnaire that can be used to evaluate psychological readiness from early phases of rehabilitation. Research on how to address inexpedient psychological readiness is underway. At current, the most important recommendations include regular monitoring to identify patients in need of particular attention. These patients should be targeted with thorough information and regular testing of physical recovery to give realistic expectations and increase motivation and confidence. Future studies should focus on achieving consensus on a psychological readiness definition, on interpretation of ACL-RSI scores, on distinguishing between helpful and inexpedient psychological responses and on how unwanted responses should be targeted.

It is time to target psychological readiness!

## Abbreviations

ACL: Anterior Cruciate Ligament
ACLR: Anterior Cruciate Ligament Reconstruction
ACL-RSI: Anterior Cruciate Ligament - Return to Sports after Injury
RTS: Return to Sport
